# Initial Validation of the Dutch Translation of the Caregiver Wish List: An Interview-Based Scale for Measuring Parenting Practices

**DOI:** 10.1177/2158244018763475

**Published:** 2018-03-09

**Authors:** Jill Thijssen, Nick J. Broers, Peter Muris, Corine de Ruiter

**Affiliations:** 1 Maastricht University, The Netherlands

**Keywords:** Caregiver Wish List, parenting practices, factor structure, validation study

## Abstract

Research has demonstrated that adequate parenting is an important determinant of a healthy social-emotional development in children. There is a need for valid assessment tools for measuring parenting quality, particularly in clinical settings. The Caregiver Wish List (CWL) is a new 53-item interview-based scale for assessing parenting practices. We examined the CWL’s factor structure in a sample of 348 parents of children (4-11 years), 220 were drawn from the general population and 128 from a clinical setting. Exploratory factor analysis revealed five factors, which did not fully correspond with the hypothesized, original factor structure. Nonetheless, the extracted factors were meaningful and could be labeled as follows: *adequate discipline, controlled responding, focus on positive behavior, consistency*, and *monitoring*. All factors demonstrated adequate internal consistency. The factor structures in community and clinical samples were comparable, supporting the generalizability of the factor structure. Furthermore, the factors differentiated between community and clinical samples, with better parenting skills observed in the community sample. Finally, all factors were significantly and negatively related to child psychopathology, with stronger correlations for externalizing than for internalizing problems. Only 23 of the 53 original CWL items loaded on at least one of the five factors, indicating that the original CWL can probably be reduced in length without losing important content. Future research needs to examine if the extracted CWL factors are sensitive to change. The CWL’s interview format provides opportunities for rapport building between parent and interviewer, and for reflection on parenting skills in terms of strengths and vulnerabilities.

Ineffective parenting is the most widely studied and empirically established risk factor for externalizing behavior problems in children (DeBaryshe, Patterson, & Capaldi, 1993; Forgatch, Bullock, & Patterson, 2004; McCoy, Frick, Loney, & Ellis, 1999; Nix et al., 1999; Oliver, Guerin, & Coffman, 2009; Patterson, Forgatch, Yoerger, & Stoolmiller, 1998; Sharma & Sandhu, 2006; Williams et al., 2009). In particular, harsh and inconsistent discipline, poor monitoring and supervision, and low positive involvement have all been demonstrated to be significantly associated with young people’s oppositional-defiant and conduct problems (Bierman & Smoot, 1991; Cunningham & Boyle, 2002; Ehrensaft et al., 2003; Frick et al., 1992; Nicholson, Fox, & Johnson, 2005). Many interventions for childhood behavior problems focus on increasing effective parenting and decreasing the use of ineffective rearing practices. Research generally supports the theoretical notion that improving parenting practices is the most important mechanism contributing to a decrease in children’s behavior problems (DeGarmo & Forgatch, 2005, 2007; Forgatch & DeGarmo, 1999; Kazdin, 2007; Martinez & Forgatch, 2001; Ogden & Amlund-Hagen, 2008). Improvements in the behavior problems of the children also tend to reduce stress and depression in parents, which in turn will increase the likelihood that the effective parenting practices will be maintained, thereby promoting the long-term effects of therapy (DeGarmo, Patterson, & Forgatch, 2004).

Many parent training programs are based on the Social Interaction Learning (SIL) model (Forgatch et al., 2004). The SIL model assumes that the psychosocial development of the child is directly influenced by the parents and their parenting strategies. Contextual factors, however, can have a negative impact on the parenting quality, and thus, indirectly on the child. For example, when parents are going through a divorce, this puts pressure on the parenting quality (Conger, Patterson, & Ge, 1995; Hetherington, Bridges, & Insabella, 1998). According to the SIL model, there are five core effective parenting practices: skill encouragement, discipline, monitoring, problem solving, and positive involvement. *Skill encouragement* refers to the enhancement of a prosocial development by using scaffolding techniques (e.g., breaking behavior into small steps, prompting appropriate behavior) and the provision of positive reinforcement (e.g., giving compliments when the child behaves well; Forgatch & DeGarmo, 1999). *Discipline* decreases problem behaviors by the appropriate and consistent use of mild sanctions, like giving a time out or taking away privileges (Patterson, 1986). *Monitoring* involves knowing the child’s friends and keeping track of the child’s activities to protect him or her against the negative influence of deviant peers (Snyder, 2002). *Problem solving* helps families to negotiate during arguments, to clearly determine rules and consequences when the rules are violated (Forgatch, Patterson, & DeGarmo, 2005). And finally, *positive involvement* concerns the many ways in which parents give their child loving attention and engagement in joint fun activities (Forgatch & DeGarmo, 1999; Forgatch et al., 2005).

Many studies rely on observations of parent–child interactions as a method to assess parenting skills (Martinez & Forgatch, 2001; Ogden & Amlund-Hagen, 2008; Patterson, DeGarmo, & Forgatch, 2004). For instance, parent and child can be observed during a structured playroom session or in a natural situation at home or in school. Self-report measures can be used in addition to observation measures of parenting. In fact, there are indications that a multi-method assessment of parenting possesses incremental validity over using only one measurement method (Harvey, Danforth, Ulaszek, & Eberhardt, 2001; Meyer et al., 2001).

There are many self-report measures that can be used for the assessment of parenting, although there is a wide variety in terms of definition and theoretical framework. The Caregiver Wish List (CWL; Hodges, 2002) is a structured interview-based instrument that enables parents and other caregivers to reflect on their own parenting practices and talk about their experiences with parenting. Interestingly, the CWL was designed to measure the core parenting practices of the SIL model and seems to be particularly useful in clinical practice for a number of reasons. First, the CWL may help clinicians to gather information on the strengths and weaknesses in parenting practices as perceived by parents themselves. Furthermore, the CWL can be expected to enhance the therapeutic alliance and the positive engagement between parents and the clinician. Finally, the CWL may assist in identifying and clarifying the goals of treatment. The goal of the CWL is to empower parents by encouraging them to see themselves as the main agent of change for their child (Hodges, 2005). The CWL contains six a priori domains of parenting skills: providing direction and following up, encouraging good behavior, discouraging undesirable behavior, monitoring activities, connecting positively with the child, and problem solving orientation.

In the Netherlands, the CWL is being used as an intake assessment for Parent Management Training–Oregon model (PMTO), a parent training program that teaches parents effective parenting skills according to the SIL model with the aim of reducing externalizing behavior problems in children aged 4 to 12 years. This makes sense as the CWL directly measures the parenting skills that are the main focus of change in PMTO. A Dutch translation of the CWL was also employed in a large-scale study evaluating the effectiveness of PMTO in The Netherlands (Thijssen, Vink, Muris, & de Ruiter, 2017). However, in that study, it was found that the reliability of the CWL was rather poor, with only one of the six original parenting domains showing adequate internal consistency. In fact, to our knowledge, no study can be found that examined the psychometric properties of the CWL. The aim of the present study was a first attempt to fill this gap. The CWL was administered in a large sample of parents of children from the community as well as a clinical setting visited by parents of whom children displayed disruptive behavior problems. In this way, it became possible to explore the factor structure of the instrument as well as its reliability (internal consistency and correlations between factors) and validity (relations between parenting scores and children’s psychopathological symptoms).

## Method

### Participants

The total sample consisted of 348 parents (92.1% mothers) of 4- to 11-year-old children (*M* age = 8.07 years, *SD* = 1.61). Of these children, 185 (53.2%) were boys and 163 (46.8%) were girls. The mean age of the main caregiver was 39.06 years (*SD* = 5.15). The majority of the main caregivers was the biological parent of the child (92.8%), had the Dutch nationality (89.1%), and was employed (72.4%). Of the 348 main caregivers, 222 (63.8%) were living with a partner. Seventy-three percent of the main caregivers had a college or university degree, 17.9% had completed high school, while 1.1% had only finished elementary school. In Table 1, the demographic characteristics are reported for the community and clinical sample separately. In comparison with the clinical sample, children and parents in the community sample were significantly older, *t*(346) = 7.94, *p* < .01, and *t*(327) = 4.95, *p* < .01, respectively. Moreover, the community sample contained more girls, χ^2^(1) = 16.00, *p* < .01, and more two-parent households, χ^2^(1) = 8.57, *p* < .01, than the clinical sample.

**Table 1.** Demographic Data and Significant Differences for the Community and Clinical Sample.

	Community (*n* = 220) *M* (*SD*) or %	Clinical (*n* = 128) *M* (*SD*) or %	*p*
Child age	8.58 (1.27)	7.18 (1.75)	.00
Parent age	40.11 (4.42)	37.00 (5.83)	.00
Child is a boy	45.0	67.2	.00
Main caregiver is female	93.6	89.1	.21
Biological parent	96.8	98.2	.64
Two-parent household	89.1	77.0	.00
Dutch nationality	92.7	94.6	.74
Employment	77.7	76.4	.79

### Procedure

The parents of the community sample were recruited in elementary schools where information letters explaining the nature of the study and consent forms were distributed, together with a brief survey to obtain background information on the parents (e.g., ethnicity, educational level, employment status). When parents agreed to participate, they signed the consent form, filled out the demographic characteristics survey, and returned these materials to the researchers via the child’s teacher at school. The parent who spent most time with the child (main caregiver) was contacted to make an appointment for the CWL interview. The CWL interview was administered at the child’s school and lasted for about 30 min. As a reward, parents received a €10 gift voucher. This study was approved by the Medical Ethical Committee of the Academic Hospital of Maastricht University (NL19855.068.07/MEC 07-3-091).

The parents of the clinical sample were recruited as part of a study on the effectiveness of PMTO in children with disruptive behavior problems. As soon as families were referred to the child service agency, it was checked whether they met the inclusion criteria for the study (for details, see Thijssen et al., 2017). Families who met the criteria received information about the study and its procedure and were invited to participate. When parents agreed, they were asked to give their written consent. The CWL interview was completed by the main caregiver and mostly took place at the child service agency. For the present study, only the CWL data that were obtained prior to the treatment were employed.

### Instruments

As noted in the introduction, the CWL (Hodges, 2005; Hodges, de Ruiter, & Thijssen, 2009) is an interview-based instrument consisting of 53 items questioning the parent about his or her parenting skills. The interviewer reads the questions to the parent, who has to indicate the most applicable response option using a 5-point Likert-type scale. The response options are specific to the question, although they mostly refer to the frequency of behaviors. The parent also has the option to respond with “not applicable” when the described situation does not apply to their family. Twenty-nine items are positively formulated, whereas 24 items are negatively phrased. The latter items are reversed so that higher CWL scores reflect higher levels of effective parenting. On the basis of conceptual work by Hodges (2002), items can be allocated to six a priori domains of parenting skills: providing direction and following up (four items), encouraging good behavior (five items), discouraging undesirable behavior (24 items), monitoring activities (13 items), connecting positively with child (three items), and problem solving orientation (four items). Each domain score can be regarded as a dimension with weak parenting skills on one end and strong parenting skills on the other.

The *Child Behavior Checklist* (CBCL) is a widely used rating scales for assessing behavioral and emotional problems of children aged 6 to 18 years (Achenbach, 1991; Dutch version: Verhulst, van der Ende, & Koot, 1996). Each scale consists of 120 items scored on a 3-point Likert-type scale (0 = *not true*, 1 = *somewhat or sometimes true*, 2 = *very or often true*). For the present study, only the internalizing and externalizing scales were used. The CBCL has good reliability and validity (Achenbach, 1991; Verhulst et al., 1996).

### Data Analyses

As no research on the factor structure of the CWL has been published, a confirmatory factor analysis was considered as less appropriate. Instead, we performed an exploratory factor analysis (EFA), using the preimposed conceptual structure as a guide in the process of exploration. Exploratory factor analyses were conducted with Mplus (version 5.21), using robust maximum likelihood estimation (MLE) to correct for the nonnormality in the distribution of the items. An oblique rotation method (geomin) was applied, as it seemed plausible that the extracted factors of positive parenting would be correlated. Unlike principal components analysis, EFA does not permit the quantification of the proportion of common variance explained. For an alternative indication of the quality of our factor solution, we decided to examine a few model fit indices that are usually reported in the context of confirmatory factor analysis: the Tucker–Lewis Index (TLI), the root mean square error of approximation (RMSEA), and the standardized root mean square residual (SRMR). These fit indices give complementary information. Although they should be considered with caution when conducting EFA, they provide a useful impression on the quality of the factor solution. Fit values that would indicate an accurate description of the original correlation matrix would be a TLI value larger than 0.95, a RMSEA value smaller than 0.05, and an SRMR value smaller than 0.08 (Hu & Bentler, 1999).

Even though the separate samples were relatively small in comparison with the number of items to be processed in the factor analysis, we decided to initially explore the factor structure for each of the samples separately. Using smaller samples in the factor analysis will increase the instability of the solution, but prevents possible confounding because of using very heterogeneous populations. Only in case of sufficient similarity in the factor solutions for the separate samples, it seemed justified to interpret the analysis for both samples combined.

To examine whether the CWL factors were significantly able to discriminate between the community and clinical sample, independent-samples *t* tests were performed. The internal consistency of the extracted factors was examined using Cronbach’s alpha. Convergent validity was assessed by means of correlational tests between the CWL factors and CBCL. An adapted version of Steiger’s (1980) formula was used to conduct tests for comparing correlation coefficients (Lee & Preacher, 2013), to examine whether there were significant differences in the correlations between CWL factors and internalizing and externalizing problems.

## Results

### Preliminary Considerations

Before conducting the factor analysis, some preliminary decisions had to be made. First, the issue of “not applicable” responses in the CWL had to be resolved. As noted earlier, parents had the option to select this response in case a given situation did not occur in daily life. We decided to treat these “not applicable” responses as missing values. As we used robust maximum likelihood estimation for our explorative factor analysis, all cases with missing values on some of the items could be retained in the analysis. Meanwhile, items that generated a relatively high (>10%) percentage of “not applicable” responses were removed from the analysis. The threshold value of 10% was chosen because until this percentage is reached, most of the traditional methods for dealing with missing values produce fairly similar results (e.g., see Barzi & Woodward, 2004). Based on this criterion, three items were removed.

Second, we screened all item distributions to identify items that were excessively skewed. Items for which a score of 4 or 5 was given in 95% of the cases in one sample and at least 90% of the cases in the other were excluded from the analyses, because it can be assumed that these items had little discriminatory power. There were six items showing such extreme distributions in both samples, which were, therefore, excluded. Furthermore, we noticed a number of items that had conspicuously low mean scores, indicating that the pertinent behaviors were hardly practiced by the parents. One would expect that the mean scores on parenting skills in the community sample would at least be “neutral” (i.e., somewhere around the rating of 3 or higher). Bearing this in mind, we excluded all items with a mean value lower than 2.50 in the community sample (Items 19, 21, 23, 24, 38, and 40). A mean below 2.50 indicates a clear majority of “almost never” or “now and then” responses (with “now and then” reflecting a lower frequency than “sometimes,” which is coded as 3 in the questionnaire). Of course, a relatively low item mean for the general community sample need not by itself invalidate the item as a diagnostic marker, as it is conceivable that the corresponding item mean might still be significantly lower in the clinical sample. However, with one exception (Item 21), the items that were singled out for exclusion on the basis of this first criterion all had higher means in the clinical sample than in the general sample. This indicates that parents from the clinical sample show the desired behavior more often than parents from the general population sample. Therefore, these items cannot be used for the desired diagnostic purpose. Item 21 had a lower mean in the clinical sample, but it did not differ significantly from that in the general population sample. We therefore decided to exclude these six items, because they do not differentiate between general population and clinical population samples. Using these three criteria, 13 items were ultimately removed before conducting the EFA.

### EFA

The Kaiser–Meyer–Olkin (KMO) measure of sampling adequacy showed acceptable values for both the community sample (KMO = 0.67) and the clinical sample (KMO = 0.64). The initial attempt to conduct EFA for the community sample failed to reach convergence in the estimation of parameter values. Inspection of the output provided, showed conspicuous estimates of means and covariances involving four items. Although failure to reach a convergent solution may have several causes, the most likely explanation seemed related to low initial communality values for these four items. We therefore decided to remove these items from the analysis, after which no further problems in convergence occurred. The EFA in the community sample was then rerun with the remaining 36 items and also performed for the clinical sample. Based on the Kaiser criterion (eigenvalues > 1), 12 factors were identified for both the community and clinical sample. Obviously, this was not a useful criterion. Inspection of the scree plot suggested a five-, six-, or a seven-factor solution. Inspection of the fit indices for both the community and clinical sample pointed in the direction of either a five- or a six-factor solution for both samples. The sixth factor was difficult to interpret and, therefore, a five-factor solution seemed most appropriate. This resulted in well-interpretable factors with acceptable RMSEA and SRMR values, but rather low TLI values (see Table 2). As the pattern of factor loadings was quite comparable in both samples, we decided to rerun the analysis for both samples combined. The resulting five-factor solution produced good fit values for the RMSEA and the SRMR measures, whereas the TLI improved but was still below the acceptable limit.

**Table 2.** Fit Indices of the Five-Factor Solution for the Community, Clinical, Total Sample, and Final Factor Solution Based on the Included Items of the Total Sample.

	Fit index		
	TLI	RMSEA	SRMR
Community sample	.50	.069	.052
Clinical sample	.54	.074	.061
Total sample	.78	.049	.042
Final factor solution total sample	.87	.050	.035

*Note.* TLI = Tucker–Lewis Index; RMSEA = root mean square error of approximation; SRMR = standardized root means square residual.

In the five-factor solution for the total sample, 13 items had nonsignificant factor loadings or factor loadings less than .40 on all factors and were, therefore, not included in the final solution. The final factor loadings for each included item in the community, clinical, and total sample are shown in Table 3. Factor 1 consisted of five items and was named *adequate discipline* because all items reflect disciplining behaviors of parents after misbehavior of their child. All five items were from the original “discouraging undesirable behavior” domain. Factor 2 encompassed four items that referred to *controlled responding* when the child misbehaves (e.g., calm, neutral tone of voice). Again, all items belonged to the original “discouraging undesirable behavior” scale, except for Item 3 which came from the original scale “providing direction and following up.” Factor 3 contained four items and was labeled *focus on positive behavior.* These items were all from different original domains, but all appeared to tap parents’ responsiveness to positive, prosocial behavior of the child. Factor 4 was composed of six items reflecting parents following through after disciplining their child. Therefore, this factor was labeled *consistency*. All items were from the original “discouraging undesirable behavior” domain. The final and fifth factor was named *monitoring* and consisted of four items which were all part of the original “monitoring activities” scale. Items 17 and 45 had substantial secondary loadings, but based on the highest loadings, they were assigned to Factor 3 and Factor 5, respectively.

**Table 3.** Factor Loading for Items on the Caregiver Wish List for the Community, Clinical, and Total Sample.

Items		Community sample	Clinical sample	Total sample
Factor 1: Adequate discipline				
10	When your child misbehaves, how often do you choose to give no consequences because you think that your child is too stressed?	**.434**	.418	**.487**
11	When your child misbehaves, how often do you choose to give no consequences because you think your child may have a tempertantrum?	**.580**	.805	**.668**
13	When your child misbehaves, how often do you choose to give no consequences because you think your child may feel less loved?	**.696**	.330	**.597**
14	When your child misbehaves, how often do you choose to give no consequences because you don’t want to lose your temper?	**.749**	.444	**.630**
15	When your child misbehaves, how often do you choose to give no consequences because it would cause a problem for you (e.g., you would have to stay home)?	**.408**	.632	**.528**
Factor 2: Controlled responding				
3	When you tell your child what to do, how often are you in an unhappy, mad or frustrated mood?	**.450**	**.396**	**.422**
25	When your child misbehaves, how often do you get angry?	**.740**	**.775**	**.849**
26	When your child misbehaves, how often do you yell?	**.678**	**.746**	**.708**
29	When you correct your child/give a consequence—and your child reacts badly (yelling back, refusing)—how often do you get angry and let it show?	**.375**	**.581**	**.557**
Factor 3: Focus on positive behavior				
2	Which do you do more—telling your child what NOT to do (STOP doing a bad behavior) vs. telling your child what TO DO (start doing a good behavior)?	**.457**	.231	.514
5	When your child behaves well, how often do you praise or compliment your child?	**.490**	**.700**	.444
17	What do you do most—Praise your child for good behavior or correct your child for bad behavior?	.459	.405	.513
49	How often are you able to express positive feelings toward your child?	.529	**.410**	.437
Factor 4: Consistency				
16	When your child misbehaves, how often do you choose to give NO consequences because it is hard for you to be strict?	**.406**	**.698**	**.518**
18	When your child misbehaves, how often do you do nothing, because nothing seems to work?	.070	**.479**	**.374**
20	When your child misbehaves, how often do you say you will give consequences, but don’t follow through?	**.499**	**.673**	**.643**
30	When you correct your child/give a consequence—and your child reacts badly (yelling back, refusing)—how often do you give your child what he or she wants?	**.550**	**.787**	**.689**
31	When you correct your child/give a consequence—and your child reacts badly (yelling back, refusing)—how often do you give up what you wanted (e.g., a cleaned-up room)?	**.394**	**.431**	**.354**
32	When you correct your child/give a consequence—and your child reacts badly (yelling back, refusing)—how often do you give additional consequences, but NOT follow through?	.382	**.458**	**.457**
Factor 5: Monitoring				
41	How much do you know about your child’s playmates, friends, or kids he or she hangs out with?	**.778**	**.597**	**.668**
42	How much do you know about the families of these kids—like where they live, their phone number, and what their parents do?	**.765**	**.905**	**.907**
44	When your child goes out, how much do you know about *who* he or she will be with?	**.601**	**.634**	**.583**
45	When your child goes out, how much do you know about *what* he or she is doing and *where* he or she is going?	.351	.411	**.347**

*Note*. Boldfaced factor loadings are significant at *p* < .05.

### Reliability

The internal consistency coefficients of the five factors were calculated for the community, clinical, and total sample. As can be seen in Table 4, all Cronbach’s alphas were in the .61 to .82 range. When adopting an alpha of .70 as cutoff point, it can be concluded that in the clinical and total sample, four of the five factors displayed good internal consistency. The CWL total score had a good internal consistency in both the community and clinical sample, as well as in the total sample. In the community sample, Cronbach’s alphas were in general somewhat lower: only the factors *adequate discipline* and *monitoring* had adequate Cronbach’s alphas. Note, however, that all CWL factors consisted of a small set of items, and that alpha values above .60 for a limited item set could still be regarded as acceptable.

**Table 4.** Internal Consistencies (Cronbach’s α) of the Extracted Factors of the Community, Clinical, and Total Sample.

	Community sample	Clinical sample	Total sample
Adequate discipline	.70	.77	.76
Controlled responding	.63	.71	.71
Focus on positive behavior	.64	.65	.66
Consistency	.61	.72	.73
Monitoring	.75	.73	.76
Total score	.71	.78	.82

Pearson’s product–moment correlations among the five factors were calculated for the two samples separately and for the total sample (Table 5). Overall, the correlations were quite low. Two correlations were significant in all three samples, namely the correlation between *controlled responding* and *focus on positive behavior* and between *adequate discipline* and *consistency*. These correlations were positive, which indicates that parents who were well able to control their emotions during limit setting were also good in focusing on positive behavior of the child, and vice versa. Furthermore, parents who did not allow other factors to influence their limit setting were also more consistent, and vice versa. It was expected that especially the three factors derived from the original “discouraging undesirable behavior” domain (i.e., *adequate discipline, controlled responding*, and *consistency*) would be correlated, but this was only true for the total sample.

**Table 5.** Pearson’s Correlations Among the Five Extracted Factors for the Community, Clinical, and Total Sample.

	1	2	3	4	5
Community sample					
1. Adequate discipline	1.00				
2. Controlled responding	.03	1.00			
3. Focus on positive behavior	.03	.26**	1.00		
4. Consistency	.41**	.13	.11	1.00	
5. Monitoring	.14*	–.06	.17*	.01	1.00
Clinical sample					
1. Adequate discipline	1.00				
2. Controlled responding	.09	1.00			
3. Focus on positive behavior	–.09	.47**	1.00		
4. Consistency	.39**	.27**	.14	1.00	
5. Monitoring	.05	.09	.04	.14	1.00
Total sample					
1. Adequate discipline	1.00				
2. Controlled responding	.16**	1.00			
3. Focus on positive behavior	.09	.44**	1.00		
4. Consistency	.47**	.32**	.26**	1.00	
5. Monitoring	.15	.10	.18**	.17**	1.00

*p < .05. **p < .01.

### External Validity

As stated before, the pattern of factor loadings was roughly similar across the two samples, motivating us to reanalyze the data on the basis of the total sample, thus reducing instability in the parameter estimates. Table 6 shows the mean scores and standard deviations on the factors for both the community and clinical sample. The difference between both samples was significant for all factors and the CWL total score (with 23 items included in the factors). On all factors, parents from the community sample had a significantly higher mean than parents from the clinical sample, indicating that the parenting skills of parents in the community sample were better than those of parents in the clinical sample.

**Table 6.** Means (*SD*s) of the Community and Clinical Sample on the Extracted Factors and Their Significant Differences.

	Community sample	Clinical sample	*t*	*p*
	*M* (*SD*)	*M* (*SD*)		
Adequate discipline	22.77 (2.98)	20.33 (4.79)	5.19	.00
Controlled responding	13.63 (2.90)	11.09 (2.85)	7.89	.00
Focus on positive behavior	15.78 (2.56)	13.62 (2.57)	7.58	.00
Consistency	27.23 (2.80)	23.83 (4.95)	7.11	.00
Monitoring	17.22 (2.07)	16.01 (3.15)	3.90	.00
Total score	96.62 (7.38)	84.85 (10.86)	10.82	.00

Pearson’s product–moment correlations between the five CWL factors and CBCL Internalizing and Externalizing subscales were calculated. As can be seen in Table 7, all CWL factors and the total score were significantly and negatively correlated with both CBCL Internalizing and Externalizing scales. Relatively low, but still significant, negative correlations were found for the CWL factor *monitoring*. Compared with the correlations for the CBCL Internalizing scale, the correlations for Externalizing were higher, even significantly higher for three of the five CWL factors, namely *controlled responding, focus on positive behavior*, and *consistency*, as well as for the CWL total score (see Table 7). This result was as expected, because the assessed parenting skills are considered to be important for improving externalizing behavior problems of the child.

**Table 7.** Pearson’s Correlations Between the Five Extracted CWL Factors and CBCL Internalizing and Externalizing Scores and the Results of Tests Comparing the Strength of These Correlations.

	CBCL Internalizing	CBCL Externalizing	*p*
Adequate discipline	−.24	−.30	.13
Controlled responding	−.26	−.41	.00
Focus on positive behavior	−.29	−.40	.02
Consistency	−.23	−.34	.02
Monitoring	−.19	−.18	.43
Total score	−.38	−.52	.00

*Note*. All correlations were significant at *p* < .01. CWL = Caregiver Wish List; CBCL = Child Behavior Checklist.

## Discussion

This study was a first attempt to investigate the psychometric qualities of the CWL, an interview-based scale for measuring parenting behaviors as defined in the SIL model (Forgatch et al., 2004). EFA was performed to examine the factor structure of the CWL using the data of parents from both a community and a clinical sample of elementary school–age children. Furthermore, the internal reliability of the extracted factors was investigated and we examined whether the CWL factors showed theoretically meaningful associations with behavior problems of the parents’ children and whether they were able to differentiate between the community and clinical sample.

The EFA revealed five factors, which were named *adequate discipline, controlled responding, focus on positive behavior, consistency*, and *monitoring*. The five-factor structure provided a reasonable description of the original correlation matrix, according to two of the conventional fit indices. However, on one fit index, the fit was quite poor. The factor *adequate discipline* measures disciplining behavior of the parents when their child misbehaves. *Controlled responding* assesses parents’ responding to misbehavior of their child. For example, whether the parent stays calm and responds in a neutral tone of voice. The factor *focus on positive behavior* relates to parents’ ability to note and reinforce desirable behaviors of their child. *Consistency* reflects the tendency of parents to follow-through on their expressed consequences, despite aversive responses of their child to the sanction. Finally, the *monitoring* factor assesses parents’ knowledge of their child’s whereabouts and friendships.

The extracted factors are only partially in line with the domains proposed by the developer of the CWL. Hodges (2002) had in mind that the CWL would be composed of six domains of parenting, whereas in the present study only five factors were found. Three of these five factors, namely, *adequate discipline, controlled responding*, and *consistency*, were entirely composed of items from the original “discouraging undesirable behavior” domain. This finding was hardly surprising as the original “discouraging undesirable behavior” domain contained about half of all original CWL items, which included a rather heterogeneous set of parenting behaviors all targeting unwanted behaviors of the child. The factor *monitoring* was in keeping with the original “monitoring activities” domain, but contained only four of the original 13 items. The factor *focus on positive behavior* contained items from several CWL domains. The only domain that did not emerge in the present factor structure was problem solving. The four original problem solving items were discarded during the EFA procedure, indicating that problem solving did not emerge as a separate and homogeneous parenting factor. It seems that direct observation measures are more effective in measuring this specific parenting skill (Bullard et al., 2010; Thijssen et al., 2017). Altogether, the five factors found for the CWL in the present study correspond to four of the five core parenting practices of the SIL model (i.e., effective discipline, encouragement, positive involvement, monitoring).

Only 23 of the 53 original CWL items loaded on at least one of the five factors, indicating that the original CWL can probably be reduced in length without losing critical content. Despite the limited number of items included in the five factors, the factors showed adequate internal consistencies, with Cronbach’s alphas ranging between .61 and .77. The CWL total score, based on the items included in the factors, had good internal consistency coefficients in the samples (.71-.82). Only two correlations among CWL factors were consistently found in the community, clinical, and total sample, namely those between *controlled responding* and *focus on positive behavior* and between *adequate discipline* and *consistency*. The correlations between the other factors were quite low. Especially the correlation between *adequate discipline* and *controlled responding* was unexpectedly low, given the fact that these two factors were derived from the same original CWL domain (i.e., discouraging undesirable behavior).

The factor structure of the community and clinical samples was highly similar, and thus, the extracted CWL factor structure seemed to be applicable to parents in both populations. Meanwhile, it was found that the two samples differed in terms of absolute scores on the five extracted CWL factors and the total score in the expected direction: Parents in the community sample exhibited higher scores and hence better parenting skills than parents in the clinical sample. Finally, all factors and the CWL total score were significantly negatively related to internalizing and externalizing behavior of the child: The better the parenting practices of the parents, the lower the behavior problems. The factors *controlled responding, focus on positive behavior*, and *consistency* were significantly stronger associated with externalizing problems than with internalizing symptoms. As the CWL measures parenting practices that are assumed to be especially relevant within the context of externalizing behavior problems, these results can be taken as support for the external validity of this parenting measure.

A few limitations of our study have to be noted. First, the majority of the families consisted of two biological Dutch parents with steady employment, so it is unknown to what extent our findings can be generalized to, for example, single parents, stepfamilies, or parents from other ethnic and lower socioeconomic backgrounds. Second, the sample size was quite small for factor analysis when considering the number of items included in the original CWL. The criterion for factor analysis is that there should be at least five cases per item (Hair, Black, Babin, & Anderson, 2009). The CWL contains 53 items, which means that at least 246 participants were needed to meet this criterion. However, before the final factor analysis was run, 17 items needed to be excluded based on various criteria, which means that only 36 items were used in the analysis. Thus, a sample of 180 participants was sufficient. The community sample and the total sample met this criterion, but the clinical sample (*n* = 128) did not. Third, our study did not include an assessment of construct validity or test–retest reliability, which could have added to our knowledge of the utility of the CWL. Finally, there was a difference in the frequency of endorsement of “not applicable” responses between the community and the clinical sample. The clinical sample showed more not applicable responses in comparison with the community sample. Two possible explanations could be given for this. It could be that certain parenting situations are less common in a clinical sample than in a community sample (e.g., giving allowance) or it could be related to the person who interviewed the parents. Parents from the community sample were interviewed by trained students who were specifically instructed to avoid not applicable responses and to stimulate parents to give the best suitable answer. Parents from the clinical sample were interviewed by trained clinicians who received the same instructions. However, these parents were recruited and interviewed over a longer period of time, which might have caused drift in the adherence to of these instructions.

As the CWL in its current form seems unsuitable for research purposes, it would probably be better to rely on another, better-validated self-report measure for assessing parenting skills; for example, the Alabama Parenting Questionnaire (APQ; Essau, Sasagawa, & Frick, 2006), which has been examined in various studies (e.g., Dadds, Maujean, & Fraser, 2003; Escribano, Aniorte, & Orgilés, 2013; Shelton, Frick, & Wootton, 1996; Zlomke, Lamport, Bauman, Garland, & Talbot, 2014). However, the APQ does not measure all the parenting skills defined by the SIL model, whereas the CWL was especially developed to measure those parenting dimensions. Also, for clinical purposes, the CWL would be more suitable than the APQ. In contrast to the APQ, the CWL is an interview and was designed to help therapists gather information on strengths and weaknesses in parenting practices as seen by parents themselves, and to enhance the therapeutic alliance and positive engagement between parents and therapists (Hodges, 2005). For this purpose, an interview is more appropriate than a questionnaire. Therefore, the CWL could be a useful instrument and it may be worthwhile to further examine its psychometric properties to improve this instrument for research purposes. Items could be added to some of the factors to improve their internal consistency. Also, the factor structure found in the present study should be replicated before more definitive conclusions about the instrument’s structural validity can be drawn. Furthermore, it needs to be examined if the extracted factors of the CWL are sensitive to change. In our study evaluating the effectiveness of PMTO, in which the CWL was used as an outcome measure, only the original “discouraging undesirable behavior” domain and the CWL total score had adequate internal consistencies and these scales showed significant change in response to parent training (Thijssen et al., 2017). In addition, future research should explore whether the parenting practices assessed by the CWL predict the development or maintenance of externalizing behavior problems in children. The association between CWL self-reported parenting practices and parenting assessed on the basis of actual parent–child interactions studied in real life or in the lab should also be examined.

In conclusion, findings from our study suggest that the CWL in its current form needs to be amended to render it more suitable for research purposes. The a priori factor structure could not be replicated and several of the extracted factors need additional items to improve the CWL’s psychometric properties. Furthermore, although the CWL was designed to assess parenting skills defined by SIL theory, it failed to assess problem solving as one of the main SIL skills. Notwithstanding, the CWL’s interview format provides opportunities for rapport building between parent and interviewer, and for reflection on parenting skills in terms of strengths and vulnerabilities.
